# A Case of Perforated Peritonitis Caused by the Migration of a Single‐Puncture Gastric Wall Fixation Device Following Percutaneous Endoscopic Gastrostomy

**DOI:** 10.1002/deo2.70159

**Published:** 2025-06-10

**Authors:** Yuu Kodama, Yuji Mizokami, Hidemitsu Nishizawa, Gen Maeda, Gen Kimura, Yuzo Toyama, Shingo Asahara, Ryuji Nagahama, Hideki Sunagawa

**Affiliations:** ^1^ Department of Gastroenterology ShinTokyo Hospital Chiba Japan; ^2^ Department of Gastroenterology New Tokyo Hospital Chiba Japan

**Keywords:** endoscopy, gastropexy, gastrostomy, laparoscopy, peritonitis

## Abstract

We report an uncommon case of perforated peritonitis resulting from the migration of a single‐puncture gastric wall fixation device following percutaneous endoscopic gastrostomy. An 83‐year‐old male developed acute peritonitis 6 days post‐procedure, requiring emergency surgery. One fixation device was found embedded in the abdominal wall, and gastric perforation was identified. To our knowledge, this is the first reported case of peritonitis caused by T‐fastener migration outside the gastric wall.

## 1 Introduction

Enteral nutrition by gastrostomy is the recommended option over nasogastric tube feeding in patients requiring nutritional support exceeding 4 weeks [1]. Currently, percutaneous endoscopic techniques are preferred over direct surgical techniques due to their association with fewer complications. Gastric wall fixation has been reported to reduce the occurrence of complications [2] and has become a standard adjunct to gastrostomy procedures. Gastric wall fixation devices are broadly classified into two types: the two‐puncture fixation method (e.g., the Funada‐type), which employs two needles to fix the gastric and abdominal walls, and the single‐puncture method, represented by the T‐fastener device, which anchors a metallic T‐bar via a single needle insertion (Figure S1).

While double‐needle fixation devices are commonly used in percutaneous endoscopic gastrostomy (PEG), there are cases where their use is challenging due to limited available space. In such instances, single‐needle fixation devices provide a feasible alternative. Although peritonitis caused by early gastrostomy tube removal before fistula formation has been reported, no cases of peritonitis resulting from a single‐puncture device penetrating the abdominal cavity have been documented. We present a rare case of peritonitis caused by the migration of a single‐puncture fixation device outside the gastric wall, necessitating emergency surgical intervention (Supporting Information).

## 2 Case Report

**FIGURE 1:**
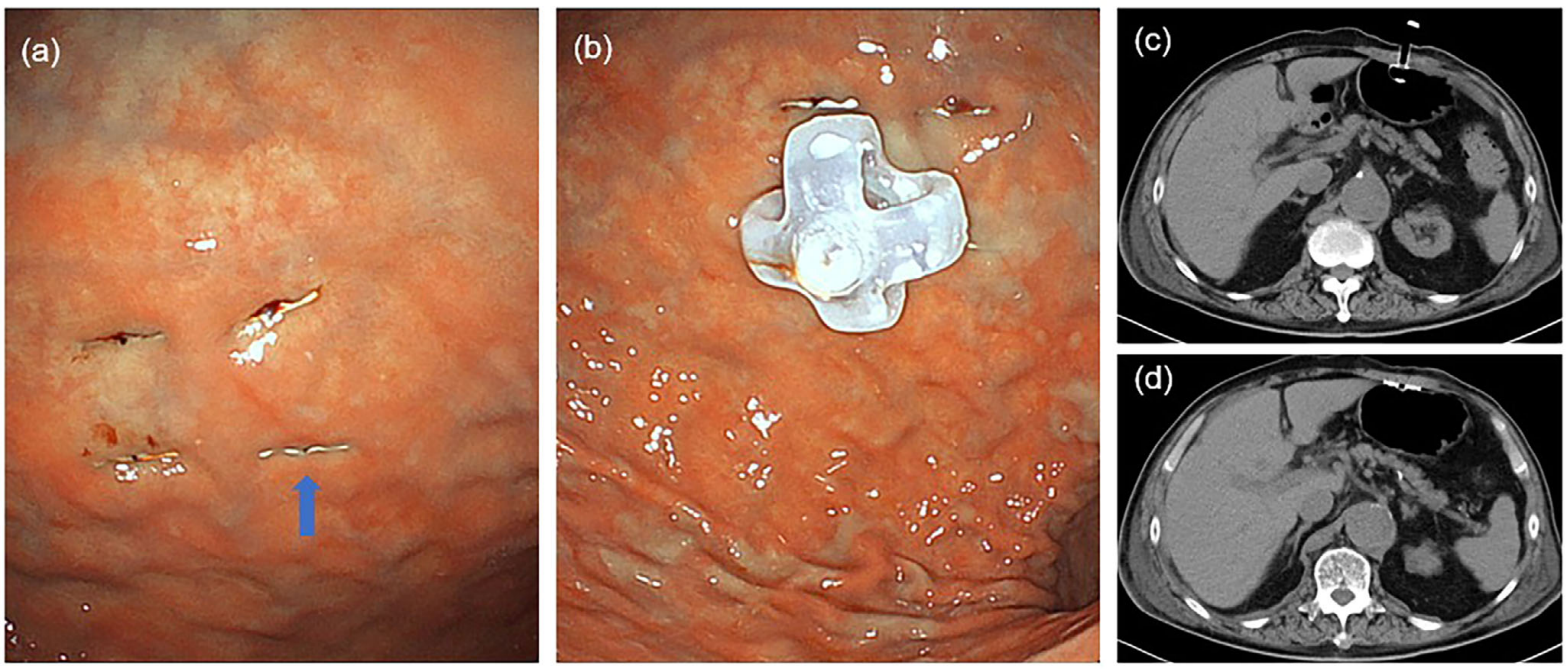
(a) Four‐point fixation was performed using single‐needle fixation devices. The fixation devices were embedded in the gastric wall. The blue arrow indicates the fixation device that later migrated into the abdominal wall. (b) A bumper‐type gastrostomy tube was placed and secured within the stomach. (c) Abdominal computed tomography (CT) immediately after gastrostomy placement. The tip of the gastrostomy tube is positioned within the stomach. (d) The abdominal wall fixation device has not migrated into the stomach.

An 83‐year‐old male with comorbidities including chronic renal failure, prostate cancer, angina, and thoracic aortic aneurysm presented, who presented with a body mass index (BMI) of 24.1, underwent coronary artery bypass grafting and ascending aortic replacement. Subsequently, he suffered multiple cerebral infarctions, he developed severe dysphagia. Despite undergoing rehabilitation therapy, he was unable to resume oral intake. At the attending physician's request, we performed an endoscopic gastrostomy with gastric wall fixation using a single‐puncture device (2‐shot anchor; Olympus, Tokyo, Japan). A bumper‐type gastrostomy tube (Ideal Button 24Fr 3.0 cm; Olympus, Tokyo, Japan) was used (Figure 1a,b). The procedure was completed without complications, and a postoperative computed tomography (CT) scan confirmed the proper placement of the gastrostomy tube and fixation devices within the stomach (Figure 1c,d). On the following day, the patient did not report any abdominal pain, and vital signs remained stable. The suture securing the fixation device was found to be embedded in the abdominal wall, accompanied by erythema and mild localized warmth; however, there was no discoloration suggestive of ischemia. As the patient remained asymptomatic with stable vital signs, enteral nutrition was resumed on the same day. Gastropexy was scheduled to be removed 7 days after the procedure. The patient's condition progressed smoothly until the sixth day post‐gastrostomy when he suddenly developed severe abdominal pain and fever. His vital signs included a temperature of 39.2°C, pulse rate of 78/min, blood pressure of 112/44 mmHg, and respiratory rate of 22/min. Physical examination revealed severe spontaneous pain, tenderness, and muscular defense throughout the abdomen. Laboratory studies showed a white blood cell count of 11,800/µL and a C‐reactive protein level of 19.3 mg/dL (Table S1), indicating a heightened inflammatory response.

**FIGURE 2:**
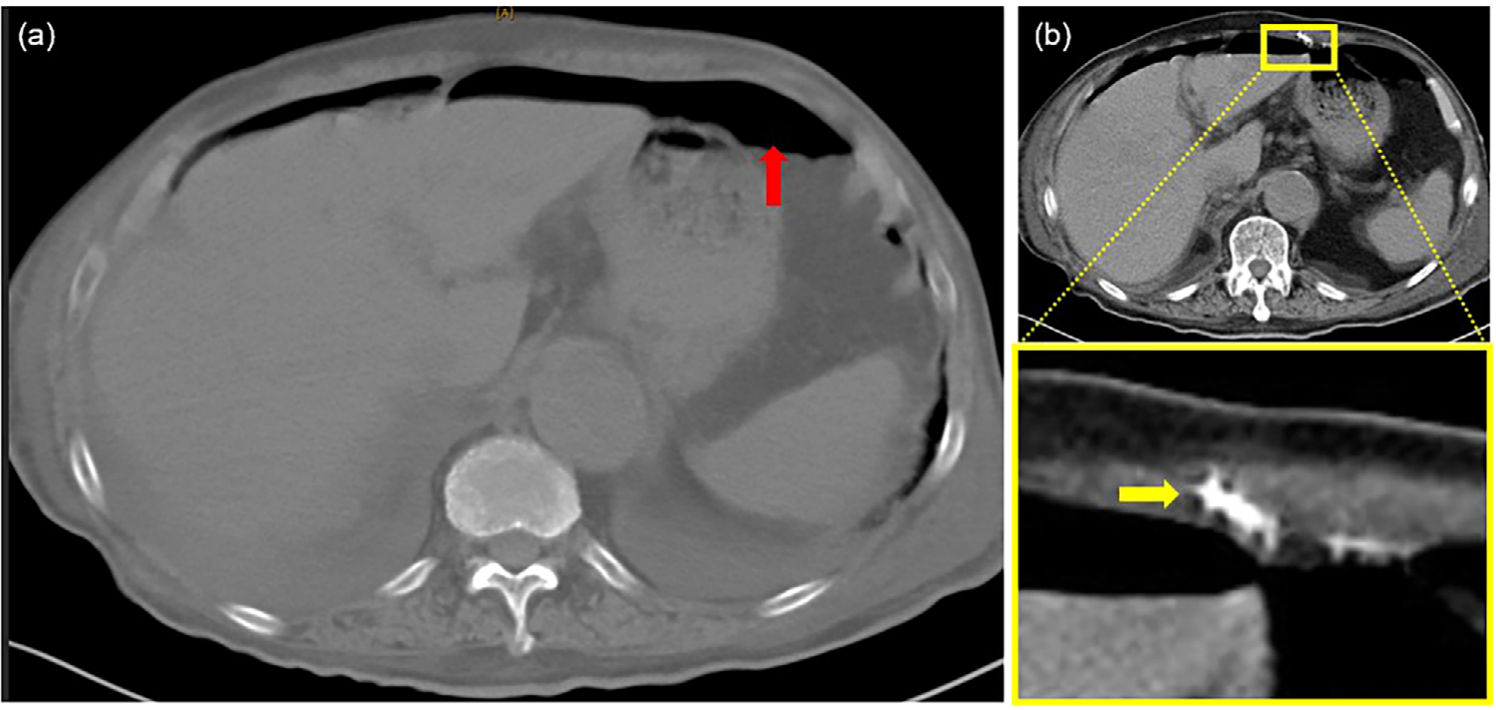
Abdominal computed tomography (CT) on day 6 After gastrostomy placement (a) A large amount of free air is observed (red arrow). (b) The gastric wall fixation device has migrated into the abdominal wall (yellow arrow).

**FIGURE 3:**
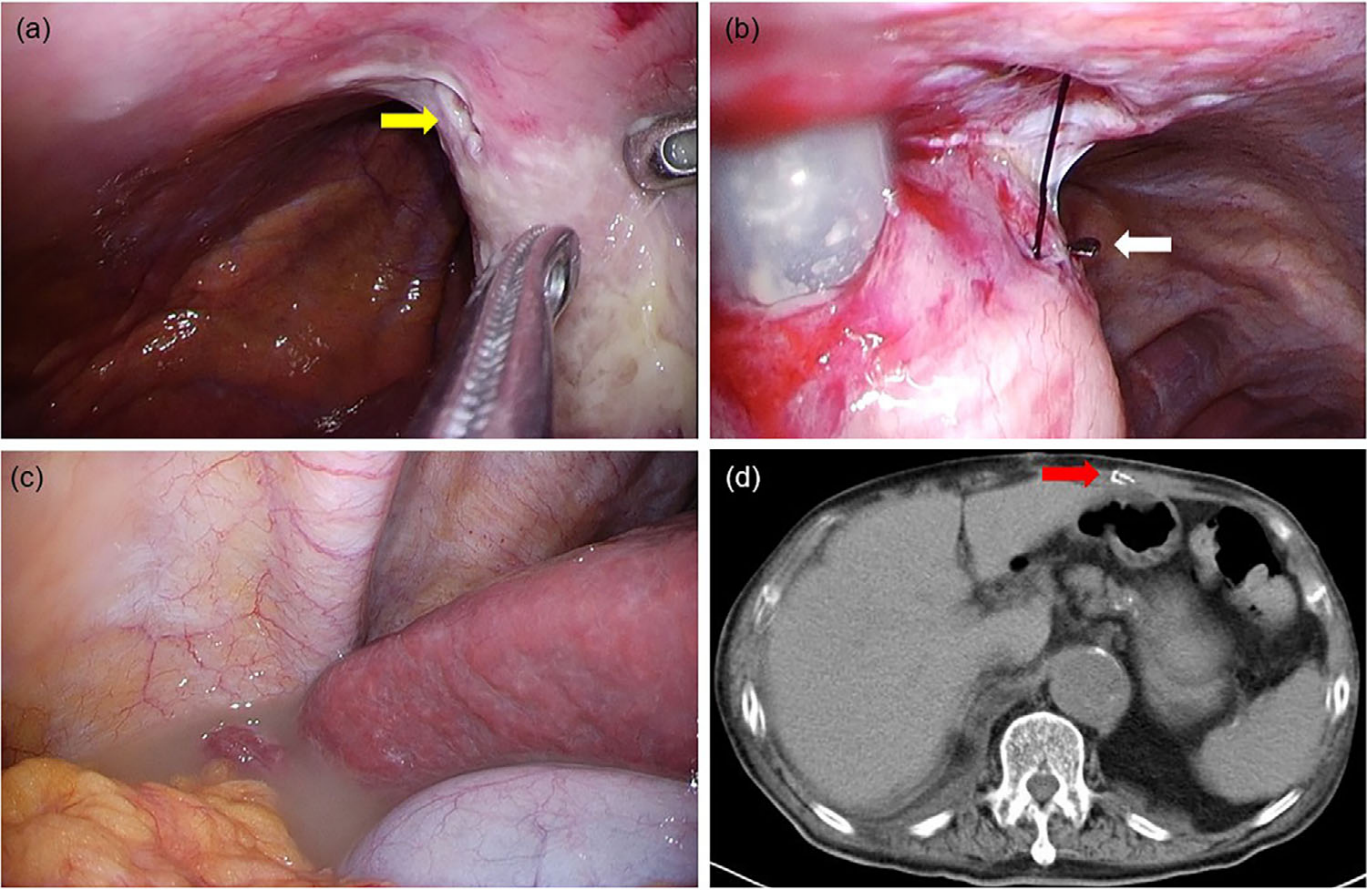
Emergency laparoscopic surgery (a) a 1 cm perforation was observed near the gastrostomy tube(yellow arrow). (b) The fixation device had deeply penetrated the gastric wall, with part of it perforating and being exposed to the abdominal cavity (white arrow). (c) Cloudy ascitic fluid was detected beneath the right diaphragm and in the pouch of Douglas. (d) Postoperative abdominal computed tomography (CT): The gastric fixation devices remained embedded within the abdominal wall (red arrow).

CT imaging revealed a significant amount of free air in the upper abdomen and considerable ascites within the abdominal cavity. One gastric wall fixation device was identified as having migrated into the abdominal wall (Figure 2). Based on a diagnosis of perforated peritonitis, emergency laparoscopic surgery was performed. During the procedure, a 1 cm perforation was observed near the gastrostomy tube (Figure 3a). Of the four gastric wall fixation devices, three were removed through a gastric wall incision, and the incision was closed with sutures. However, the remaining device near the perforation site could not be identified. Three gastric wall fixation devices had deeply penetrated the gastric wall, with parts of them perforating and being exposed to the abdominal cavity (Figure 3b). Cloudy ascitic fluid was detected beneath the right diaphragm and in the pouch of Douglas (Figure 3c); however, overall contamination within the abdominal cavity was minimal. The perforation was treated using omental patching after thorough lavage and drainage. Postoperative abdominal CT confirmed that one gastric wall fixation device had migrated into the abdominal wall (Figure 3d). The migrated device was found to have embedded into the abdominal wall, which explains why it could not be identified during the surgery. Following surgical intervention, the patient experienced no further complications related to the gastrostomy and was successfully discharged on postoperative day 84 (Figure S2).

## 3 Discussion

**FIGURE 4:**
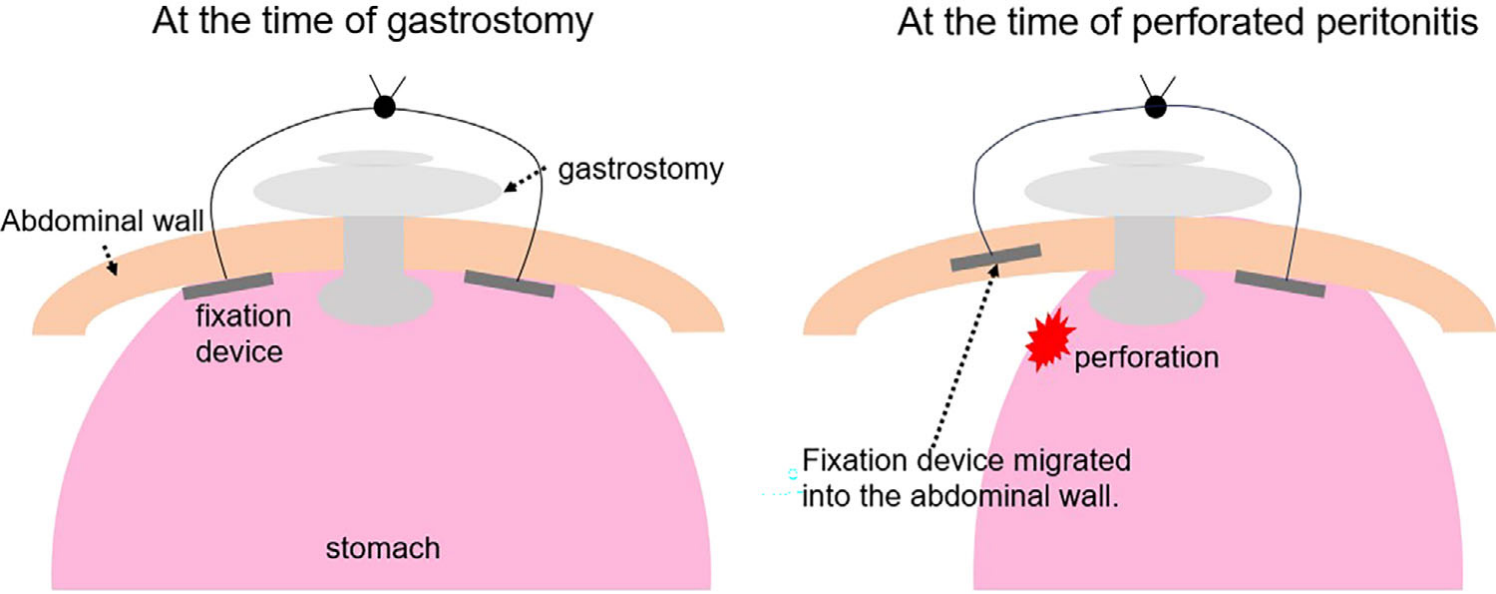
Schematic illustration showing the gastric wall fixation device, stomach, and abdominal wall.

In this case, perforated peritonitis occurred, and one of the gastric wall fixation devices was found to be embedded in the abdominal wall. Considering that the fixation device, which was initially positioned within the stomach during gastrostomy placement, had migrated into the abdominal cavity by the time peritonitis developed (Figure 4), it is presumed that the perforation occurred when the fixation device migrated outside the gastric wall. A PubMed search using the keywords “gastric wall fixation, peritonitis,” “gastric wall fixation, perforation,” “gastropexy, peritonitis,” and “gastropexy, perforation” yielded no similar reports.

Gastric wall fixation during gastrostomy is generally associated with fewer complications. It has also been reported that gastric wall fixation can prevent peritonitis in cases of accidental removal immediately after gastrostomy placement.

Gastric wall fixation devices are broadly categorized into single‐puncture fixation and double‐puncture fixation. Studies comparing these methods have not shown a significant difference in complication rates [3]. However, T‐fasteners used in single‐puncture fixation are reportedly prone to migration into the abdominal wall. Approximately one‐third of T‐fasteners migrate to the abdominal wall within 2 weeks [4], though no cases of perforated peritonitis or abscess formation due to T‐fastener migration outside the stomach have been reported, making this the first documented case.

The mechanism by which T‐fasteners migrate into the abdominal cavity remains unclear; however, a similar phenomenon, known as buried bumper syndrome (BBS), has been reported. BBS involves the migration of a gastrostomy tube outside the gastric wall due to ischemia‐induced pressure necrosis caused by mechanical pressure on the gastric wall. In this case, a review of the endoscopic images taken during the gastrostomy procedure revealed that the gastric wall fixation device was significantly embedded in the gastric wall, suggesting that mechanical pressure may have contributed to its migration outside the stomach. It has been reported that gastrostomy tubes with a smaller distal surface area increase the risk of BBS [5]. In our case, the gastric wall fixation device, which had an even smaller surface area than the gastrostomy tube, exerted pressure on the gastric wall. Therefore, the risk of gastric wall embedding was considered to be higher with the gastric wall fixation device than with the gastrostomy tube. There have been reports indicating that a Prognostic Nutritional Index (PNI) of ≤37 is associated with a higher early mortality rate and an increased risk of complications following PEG [6]. The PNI is calculated using the formula: PNI = 10 × serum albumin (g/dL) + 0.005 × total lymphocyte count (×10^9^/L of peripheral blood). In the present case, the PNI was 32.8 (Table S1), suggesting a high risk for the development of complications.

Additionally, a previous study reported that immediate removal of T‐fasteners after gastrostomy placement did not result in an increased incidence of complications [7]. In this case, physical examination revealed that the suture securing the external fixation device was embedded in the abdominal wall, which suggested that the internal gastric fixation device might also have been embedded in the gastric wall. Therefore, early removal of the fixation device should have been considered at that stage.

Even if T‐fasteners or gastrostomy tubes migrate outside the stomach, it is believed that peritonitis or abscess formation does not occur if a fistula has formed. In this case, the absence of fistula formation was likely a contributing factor. Excessive compression of the gastric wall by a gastrostomy tube has been reported to cause ischemia, necrosis, and infection, leading to an increased risk of complications [8]. In this case, although the compression was caused by the gastric wall fixation device rather than the gastrostomy tube, the excessive pressure on the gastric wall likely led to impaired blood flow. Consequently, the device not only became embedded in the gastric wall but may also have interfered with proper fistula formation. In light of this case, our department has implemented measures to avoid over‐tightening the fixation device during gastric wall fixation. In instances where excessive tension has been applied to the abdominal wall during fixation, we consider early removal of the T‐fasteners.

In conclusion, We report a rare case in which a single‐anchor gastric wall fixation device perforated the gastric wall and caused perforated peritonitis. Although this is an extremely rare complication, it is essential to consider peritonitis in the differential diagnosis when abdominal pain or worsening inflammatory markers are observed after gastrostomy placement. Early recognition and appropriate intervention are crucial to preventing severe outcomes.

## Conflicts of Interest

The authors declare no conflicts of interest

## Consent

Written informed consent was obtained from the patients for publication of this case report and any accompanying images.

## Supporting information




**FIGURE S1** Schematic diagram of a single‐needle fixation device. (a) The single‐needle fixation device is inserted while the stomach is insufflated. (b) A metallic T‐bar is deployed. (c) The device is withdrawn, leaving the T‐bar in place. (d) The device is reinserted, and a second T‐bar is deployed and left in place. (e) The device is withdrawn again, leaving the second T‐bar in place. (f) The fixation sutures are tied securely.

**FIGURE S2** The timeline of the patient's clinical course.

**TABLE S1** Laboratory data
