# Supplementary figures and images for: A Case of Perforated Peritonitis Caused by the Migration of a Single‐Puncture Gastric Wall Fixation Device Following Percutaneous Endoscopic Gastrostomy
Source: DEN Open. 2025 Jun 10;6(1):e70159. doi: 10.1002/deo2.70159 (PMC12151681; doi:10.1002/deo2.70159)

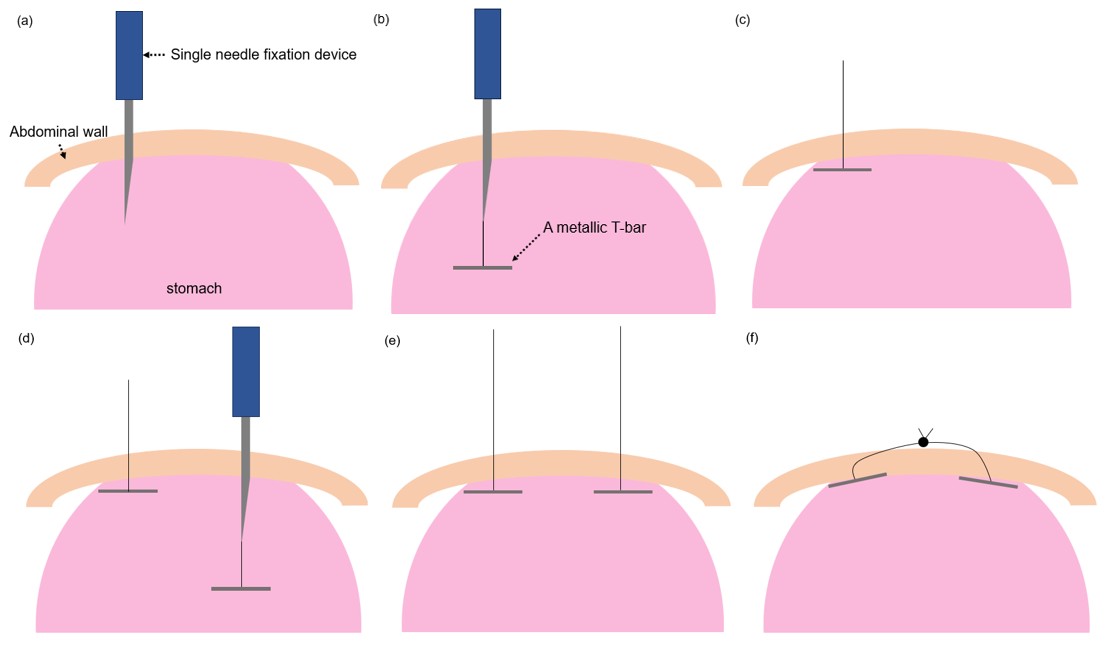

Supplement: Supplementary file 1 — FIGURE S1 Schematic diagram of a single‐needle fixation device. (a) The single‐needle fixation device is inserted while the stomach is insufflated. (b) A metallic T‐bar is deployed. (c) The device is withdrawn, leaving the T‐bar in place. (d) The device is reinserted, and a second T‐bar is deployed and left in place. (e) The device is withdrawn again, leaving the second T‐bar in place. (f) The fixation sutures are tied securely. [file DEO2-6-e70159-s002.jpg]

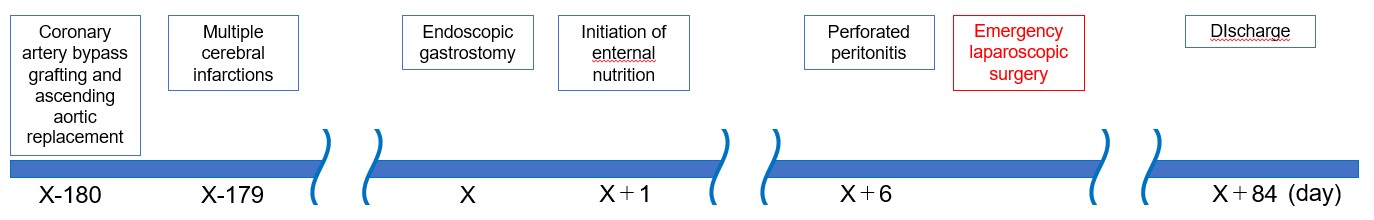

Supplement: Supplementary file 2 — FIGURE S2 The timeline of the patient's clinical course. [file DEO2-6-e70159-s003.jpg]
